# Patchouli Alcohol Modulates the Pregnancy X Receptor/Toll-like Receptor 4/Nuclear Factor Kappa B Axis to Suppress Osteoclastogenesis

**DOI:** 10.3389/fphar.2021.684976

**Published:** 2021-06-08

**Authors:** Qian Lu, Chao Jiang, Jialong Hou, Hao Qian, Feifan Chu, Weiqi Zhang, Mengke Ye, Ziyi Chen, Jian Liu, Hanbing Yao, Jianfeng Zhang, Jiake Xu, Te Wang, Shunwu Fan, Qingqing Wang

**Affiliations:** ^1^ Department of Orthopaedic Surgery, Sir Run Run Shaw Hospital, Zhejiang University School of Medicine, Hangzhou, China; ^2^ Department of Orthopaedics, The Second Affiliated Hospital and Yuying Children’s Hospital of Wenzhou Medical University, Wenzhou, China; ^3^ School of Biomedical Sciences, University of Western Australia, Perth, WA, Australia; ^4^ Department of Orthopaedics, Huzhou Central Hospital, Huzhou, China

**Keywords:** PXR, patchouli alcohol, receptor activator for nuclear factor κB ligand, osteoclast, osteoporosis, nuclear factor κB

## Abstract

The incidence of osteoporosis, which is primarily characterized by plethoric osteoclast (OC) formation and severe bone loss, has increased in recent years. Millions of people worldwide, especially postmenopausal women, suffer from osteoporosis. The drugs commonly used to treat osteoporosis still exist many disadvantages, but natural extracts provide options for the treatment of osteoporosis. Therefore, the identification of cost-effective natural compounds is important. Patchouli alcohol (PA), a natural compound extracted from *Pogostemon cablin* that exerts anti-inflammatory effects, is used as a treatment for gastroenteritis. However, no research on the use of Patchouli alcohol in osteoporosis has been reported. We found that PA dose-dependently inhibited the receptor activator of nuclear factor kappa-B ligand (RANKL)-induced formation and function of OCs without cytotoxicity. Furthermore, these inhibitory effects were reflected in the significant effect of PA on the NF-κB signaling pathway, as PA suppressed the transcription factors NFATc1 and c-Fos. We also determined that PA activated expression of the nuclear receptor pregnane X receptor (PXR) and promoted the PXR/Toll-like receptor 4 (TLR4) axis to inhibit the nuclear import of NF-κB (p50 and p65). Additionally, PA exerted therapeutic effects against osteoporosis in ovariectomized (OVX) mice, supporting the use of PA as a treatment for osteoporosis in the future.

## Introduction

Bone is remodeled under the actions of osteoclasts (OCs) and osteoblasts. The balance between these activities in bone remodeling is disrupted by an excess of bone resorption over bone formation (Feng and McDonald, 2011; Lin et al., 2020) resulting from overactivation of OC function or the presence of excessive OCs (Eastell et al., 2016). Postmenopausal osteoporosis and resulting fractures are common and devastating (Gallagher, 1990). Chronic treatment with drugs such as bisphosphonates and risedronate increases the risk of rare but acute effects, including atypical femoral fractures and osteonecrosis of the jaw (Khosla et al., 2012; Shane et al., 2014). The significance of antiresorptive therapy and risk of unwanted effects remain controversial (Black and Rosen, 2016). Natural compounds have received recent, increasing attention, and related research has continued to develop. Active plant-derived natural products are widely used for the treatment of osteoporosis (Deng et al., 2017; Wang et al., 2017; Liu et al., 2020). Therefore, we examined nature compounds with certain therapeutic effects against osteoporosis and ensured their safety and nontoxicity.

OCs are giant, multinucleated cells derived from bone marrow monocytes whose differentiation is regulated by two significant factors: receptor activator of nuclear factor κ B ligand (RANKL) and macrophage colony-stimulating factor (M-CSF) (Suda et al., 1992). Estrogen deficiency, especially in postmenopausal women, mediates RANKL generation by osteoblasts, increasing the number of new bone-remodeling units via the activation of OCs (Kanis, 1994). Tumor necrosis factor receptor-associated factor six (TRAF6), an adapter of RANKL, interacts with NF-κB-inducing kinase (NIK) to activate noncanonical (or alternative) pathways (Kobayashi et al., 2002). After its modification by the ubiquitin-conjugating enzymes Ubc13 and Uev1A, TRAF6 first binds TAK1-binding protein two (TAB2) on the cell membrane to form a complex with and activate transforming growth factor (TGF)-beta activated kinase one (TAK1), which activates inhibitor of nuclear factor kappa B kinase subunit beta (IκK) to ultimately promote the degradation of IκB and nuclear translocation of p65 (a subunit of the NF-κB dimer) (Moscat et al., 2003; Chen et al., 2006; Adhikari et al., 2007; Kawai and Akira, 2007). Normally, NF-κB associates with its inhibitor IκB in the cytoplasm and exists in an inactive state, but the inflammatory response induces dissociation of the NF-κB dimer, resulting in the nuclear translocation of p65. Therefore, the excessive activation of OCs by inflammation is also closely related to osteoporosis (Napetschnig and Wu 2013; Junren et al., 2021).

Toll-like receptor four (TLR4), which primarily regulates inflammation, has four adaptors, including myeloid differentiation primary response protein (Myd88) and TIR domain-containing adapter molecule one (TRIF), which both recruit TRAF6 (West et al., 2006; Kawai and Akira, 2007; Lim and Staudt, 2013). In pre-OCs and OCs undergoing the differentiation process, Myd88 is activated, recruiting TRAF6 to promote the NF-κB signaling pathway (Sato et al., 2004).

Pregnane X receptor (PXR), also named nuclear receptor subfamily one group I member two (Nl1r2), is a nuclear receptor activated by heterologous and endogenous chemical compounds Oladimeji and Chen, 2018). Agonists of PXR include endobiotic chemical materials, such as bile acids, pregnane, and xenobiotics, and innumerable clinical drugs used in traditional Chinese medicine (Oladimeji and Chen, 2018). PXR balances inflammatory pathways to maintain immunity (Qiu et al., 2016), primarily via blockade of the TLR4 signaling pathway and other Toll-like receptor family signaling pathways, such as TLR2 signaling (Qiu et al., 2016). PXR activation by chemical compounds involves the RANKL-induced signaling pathway and inhibits OC differentiation (Guo et al., 2017). Moreover, it’s reported that PXR/NF-κB signaling is related to Post-inflammatory Irritable Bowel Syndrome, indicating their link to inflammatory pathways (Shao et al., 2021). Although PXR and NF-κB are the core elements of the inflammatory signaling pathway, the role of their interaction in the regulation of OCs has not been clarified (Zhou et al., 2016b; Li et al., 2019).

Patchouli alcohol (PA), a tricyclic sesquiterpene extracted from *Pogostemon cablin*, has anti-inflammatory activity in gastrointestinal cell model and can regulate NF-κB pathways. (Jeong et al., 2013; Xu et al., 2017b; Zhou et al., 2018; Okamura et al., 2020). However, there have been no reports on the relationship between PA and OCs. Therefore, we investigated the anti-inflammatory effects of PA and related signaling pathways and examined the role of PA in RANKL-induced osteoclastogenesis and its molecular mechanisms. We found that PA effectively inhibits osteogenesis and OC function via the activation of PXR, inhibiting NF-κB signaling pathways. Although the relationship among PA, PXR and NF-κB was reported (Zhang et al., 2020), their interaction and effects on OCs were under investigation. In our study, molecule docking and other experiments have been applied to uncover the function and mechanism of PA suppressing osteoclastogenesis.

## Materials and Methods

### Materials and Reagents

Alpha-modified minimal essential medium (α-MEM), penicillin/streptomycin (P/S), and fetal bovine serum (FBS) were acquired from Thermo Fisher Scientific (Carlsbad, CA, United States).

PA purchased from Chengdu Herb Purify Co., Ltd. (Chengdu, China) was dissolved in dimethyl sulfoxide (DMSO) to form a 100 mM stock solution. Then, the PA solution was diluted with α-MEM and used for cell culture (Lian et al., 2019).

M-CSF and GST-rRANKL were purchased from R&D Systems (Minneapolis, MN, United States) and used in the experiments in this study according to previous literature (Ladner et al., 1988; Xu et al., 2000).

TRIzol and rhodamine-conjugated phalloidin were obtained from Thermo Fisher Scientific (San Jose, CA, United States). Cell Counting Kit-8 (CCK-8) was acquired from Engreen Biosystem (Beijing, China). A TRAcP staining kit was obtained from Sigma-Aldrich (Sydney, NSW, Australia). Reagents for the luciferase assay and oligo-dT primers were purchased from Imgenex (Littleton, CO, United States). Small interfering RNA (siRNA) for RNA interference was purchased from GenePharma (Shanghai, China). Specific antibodies against NFATc1, integrin β3, cathepsin K, vacuolar (H)-ATPase-d2 (V-ATPase-d2), histone H3 and β-actin were obtained from Santa Cruz Biotechnology (San Jose, CA, United States). Specific antibodies against TLR4, Myd88, TRAF6, PXR, p65, p-p65, and IκB were purchased from Cell Signaling Technology (Beverly, MA, United States).

### Cell Culture and Cytotoxicity Assays

The femurs and tibias of 6 week-old C57BL/6 mice were used to acquire bone marrow-derived macrophages (BMMs). Wenzhou Medical University approved all relevant procedures. Complete α-MEM containing 1% (v/v) P/S, 10% (v/v) FBS, and 25 ng/ml M-CSF was used for the cultivation of BMMs.

For the cytotoxicity assay, BMMs were first plated in 96-well plates at 7 × 10^3^ cells/well. The BMMs were then incubated in complete α-MEM containing 25 ng/ml MCSF for 24 h and then incubated with PA at different concentrations for another 48 h until the cells converged. The BMMs were incubated with 10 μL of CCK-8 solution for another 2 h, after which the absorbance at 450 nm was evaluated and measured with a microplate reader (Multiskan Spectrum; Thermo LabSystems, Chantilly, VA, United States).

### TRAcP Staining

BMMs were plated in 96-well plates at a concentration of 7 × 10^3^ cells/well and cultivated overnight to adapt to the environment. Passage-one BMMs were treated with GST-rRANKL (50 ng/ml) in the presence or absence of PA. The complete medium was exchanged for medium containing fresh GST-rRANKL and PA every 2 days until the sixth day. The cells were then washed in phosphate-buffered saline (PBS) and fixed with 2.5% glutaraldehyde for 15 min for TRAcP staining. All TRAcP-positive cells containing more than three nuclei were deemed OCs.

### Bone Resorption Assay

To measure the demineralization function of OCs, a hydroxyapatite resorption assay was performed. BMMs were plated in 6-well collagen-coated plates at 1 × 10^4^ cells per well. The medium was replaced with medium containing fresh M-CSF (25 ng/ml) and GST-rRANKL (50 ng/ml) every 2 days to facilitate the formation of OCs. The cells were extracted and transferred to the wells of a hydroxyapatite-coated plate or bone slice (CLS3989, Corning, NY, United States) at 7 × 10^3^ cells per well. Complete α-MEM containing fresh M-CSF (25 ng/ml) and GST-rRANKL (50 ng/ml) with or without PA was used to culture mature OCs for 2 days. After bleaching for 10 min and removal of the cells from the wells, bone resorption area was measured. Microscopy was used to capture images of the resorbed areas, and ImageJ software was used to analyze the OC resorption area.

### Quantitative Real-Time Polymerase Chain Reaction Analysis

BMMs at 2 × 10^4^ cells per well were plated in a six-well plate and treated with GST-rRANKL (50 ng/ml) and M-CSF (25 ng/ml) in the presence or absence of PA at various concentrations for 5 days. Total RNA was extracted using TRIzol. An oligo-dT primer was used to reverse transcribe single-stranded cDNA from 2 μg of total RNA. Real-time PCR amplification of the resulting cDNA was performed using SYBR Green (Imgenex, Littleton, CO, United States) and specific primers. The expression levels of target genes were normalized to expression of the housekeeping gene Hprt. The Livak equation was used to calculate the fold change in expression and ratio of expression in the experiment group compared to the vehicle group. The sequences of all the primers used are listed in Table 1.

**TABLE 1:** Primer sequences used for qRT-PCR.

Gene	Forward (5′→3′)	Reverse (5′→3′)
Nfatc1	GGA​GAG​TCC​GAG​AAT​CGA​GAT	TTG​CAG​CTA​GGA​AGT​ACG​TCT
C-fos	GCGAGCAACTGAGAAGAC	TTGAAACCCGAGAACATC
Acp5	TGTGGCCATCTTTATGCT	GTCATTTCTTTGGGGCTT
Ctsk	CCA​GTG​GGA​GCT​ATG​GAA​GA	AAG​TGG​TTC​ATG​GCC​AGT​TC
PXR	TCA​AGG​ATT​TCC​GGC​TGC​G	GTA​GGT​TGA​CAC​ATC​GGC​CA
TLR4	AAT​CCC​TGC​ATA​GAG​GTA​GTT​CC	ATC​CAG​CCA​CTG​AAG​TTC​TGA
Hprt	GTT​GGG​CTT​ACC​TCA​CTG​CT	TAA​TCA​CGA​CGC​TGG​GAC​TG

### Western Blot Analysis

To initially evaluate the impact of PA on functionally related proteins and major marker genes, BMMs at 1 × 10^5^ cells per well were transferred to six-well plates and stimulated with RANKL with or without PA (10 μM) for 5 days. The cells were lyzed to extract proteins in radioimmune precipitation assay (RIPA) lysis buffer (100 g/ml PMSF, 500 g/ml DNase I and phosphatase inhibitors) after 0, 1, 3 and 5 days. To examine short-term signaling pathways, BMMs (2 × 10^5^ cells per well) were seeded in six-well plates and incubated in complete medium containing 25 ng/mL M-CSF overnight to adapt to the environment. Under starvation treatment for 4 h the following day, the cells were pretreated with PA for 2 h and then stimulated with 50 ng/ml RANKL. Proteins were extracted using RIPA lysis buffer at 0, 10, 20, 30, and 60 min. The protein mixture was loaded on 10% sodium dodecyl sulfate-polyacrylamide gel electrophoresis (SDS-PAGE) gels for protein separation, and the proteins were transferred onto PVDF membranes (Bio-Rad, Hercules, CA, United States). The membranes were blocked in 5% skim milk for 1.5 h and incubated overnight at 4°C with primary antibodies. The membranes were then washed with Tris-buffered saline/Tween (TBST) three times for 15 min and incubated with horseradish peroxidase (HRP)-conjugated specific secondary antibodies for 1.5 h. The membranes were processed with enhanced chemiluminescence reagents (Amersham, Piscataway, NJ, United States) according to the manufacturer’s instructions, and images were taken using an ImageQuant LAS 4000 (GE Healthcare, Sydney, NSW, Australia).

### Luciferase Reporter Gene Assay

BMMs were cultured in a 48-well plate at 2 × 10^4^ cells per well. The cells were transiently cotransfected with 1 μg of pGL6-NF-κB-Luc plasmid using Lipofectamine 3000 (Invitrogen, Carlsbad, CA, United States) and cultured overnight to adapt to the environment before pretreatment with PA at various concentrations (1, 2.5, 5, and 10 μmol L^−1^) for 1 h, followed by stimulation with RANKL for another 6 h. The cells were harvested for luciferase activity analysis, and luciferase activity was normalized to internal control activity.

### Immunofluorescence Staining for F-Actin and p65

BMMs at 7 × 10^3^ cells per well were transferred to a 96-well plate and incubated with complete α-MEM containing 25 ng/ml M-CSF overnight. The cells were stimulated with 50 ng/ml RANKL in the presence or absence of PA for five days. The BMMs were fixed in 4% paraformaldehyde (PFA) for 10 min, blocked with 3% bovine serum albumin (BSA) in PBS for 30 min and probed with rhodamine-conjugated phalloidin (Thermo Fisher Scientific) for 45 min to stain F-actin. The BMMs were incubated with primary anti-p65 antibody and secondary antibody (Sigma-Aldrich, Australia) conjugated to Alexa Fluor-488 (Beverly, MA, United States) for another 2.5 h. 4′,6-Diamidino-2-phenylindole dihydrochloride (DAPI) (Santa Cruz, CA, United States) treatment for 10 min was used to stain the cell nuclei, and the results were visualized using a confocal fluorescence microscope (Nikon, A1 PLUS, Tokyo, Japan).

### Computational Docking

We predicted possible binding between proteins and our drug using BIOVIA Discovery Studio Visualizer (2016) (Waltham, MA, United States). Protein structure data were first obtained from the PBD website (https://www.rcsb.org/), with structures obtained by X-ray diffraction preferred. The PubChem website (https://pubchem.ncbi.nlm.nih.gov/) was searched for PA, and its two-dimensional structure was downloaded as an SDF file. After the removal of ions, unrelated structures and elements, the active site of the protein was identified using the semiflexible LibDock operation, and the potential to bind PA was analyzed. The result was used to analyze nonbonding interactions between PA and the protein, and hydrogen bonds between PA and neighboring amino acid residues in the proteins were analyzed.

### Cell Transfection

BMMs at 7 × 10^3^ cells/well were transferred to 96-well plates for overnight culture. The cells were transfected with siRNAs (20 µM) (GenePharma, Shanghai, China) using the siRNA transfection reagent GP-transfect-Mate (GenePharma) based on the specific instructions from the manufacturer.

The cells were incubated with a siRNA mixture (100 nm) for 5 h and cultured in α-MEM containing 10% FBS. mRNA expression levels after 48 h and protein expression levels after 72 h were detected to assess the results of cell transfection. The transfected BMMs were stimulated with M-CSF (25 ng/ml), RANKL (50 ng/ml) and PA (10 µM) for further experiments.

### Determination of mRNA Stability

BMMs were stimulated with 50 ng/ml RANKL for 60 h and then treated with actinomycin D (5 μg/ml) (Sigma, St. Louis, MO, United States) to stop mRNA transcription. The cells were collected, and total RNA was extracted at designated time points (after 0, 1, 2, 3, and 5 h of actinomycin D treatment) to assess mRNA degradation levels. The transcription of target mRNAs was analyzed using qRT-PCR.

### Mouse Ovariectomy Procedure

The Institutional Animal Ethics Committee of Wenzhou Medical University approved all *in vivo* experiments (ethical approval No. wydw 2019–0247). Female 12 week-old C57BL/6J mice were obtained from the Animal Center of the Chinese Academy of Science (Shanghai, China) and randomly divided into three equal groups: the sham group, ovariectomized (OVX) group, and OVX + PA group (10 mg/kg). PA was dissolved in saline containing 5% DMSO (Leong et al., 2019). The mice were housed in separate ventilated cages in a specific-pathogen-free (SPF) room at five rats per cage. The mice were adapted to this environment for 7 days prior to surgery. All mice in the sham group received sham operation + vehicle (normal saline; NS), and mice in the OVX and OVX + PA groups underwent OVX to create a mouse model of estrogen deficiency-induced osteoporosis (OVX mouse model) according to a previous study(Thompson et al., 1995; Zhou et al., 2016a). Seven days later, mice in the OVX + PA group received PA (10 mg/kg) by intraperitoneal injection, and those in the sham and OVX groups received PBS at the same volume every 2 days for 6 weeks. The mice were euthanized, and the tibias from both sides were removed for histological and micro-computed tomography (μCT) analyses.

### Micro-CT Scanning

The tibias were fixed in 4% PFA for 24 h and analyzed using a micro-CT instrument (SkyScan 1,176; Bruker, Kontich, Belgium). The images were processed using an adequate scanning protocol based on a previous study with the following settings: an X-ray tube voltage of 50 kV, a source current of 500 μA, an isotropic pixel size of 9 μmol L^−1^, and an aluminum filter thickness of 0.5 mm (Zhou et al., 2016a). NRecon Reconstruction software was used to reconstruct the images.

Areas with a volume of 0.5 mm below the growth plate and a height of 1 mm were recommended for analyses, and bone loss was estimated from the data by determining the bone volume/tissue volume ratio (BV/TV), trabecular thickness (Tb.Th), trabecular number (Tb.N), and trabecular separation (Tb.Sp).

### Histological and Histomorphometric Analyses

Tibias were fixed in 4% PFA for 48 h, and the right tibias of mice in the different groups were decalcified by treatment with 14% EDTA at 37°C for 10 days. The paraffin-embedded tibias were sagittally sectioned at a 5 μm thickness using a microtome, and the sections were stained with hematoxylin and eosin (H and E) and anti-TRAcP antibody. A uScope MXII digital microscope slide scanner (Microscopes International, Lubbock, TX, United States) was used to scan and process the section images. The OC number per bone surface (N.Oc/BS) was calculated using BIOQUANT OSTEO 2011 software.

### Statistical Analysis

All of the obtained values are presented as the mean ± standard deviation (SD). One-way ANOVA plus Tukey’s test or Kruskal-Wallis analysis (non-parametric ANOVA) plus Dunn’s multiple comparison test (when the data failed the assumptions of one-way ANOVA) were used to test the significance of differences between groups. Two-way ANOVA was performed to examine the effects of time and treatments on protein expression levels. Statistical significance was indicated when *p* < 0.05.

## Results

### Patchouli Alcohol Affected Osteoclastogenesis

**FIGURE 1:**
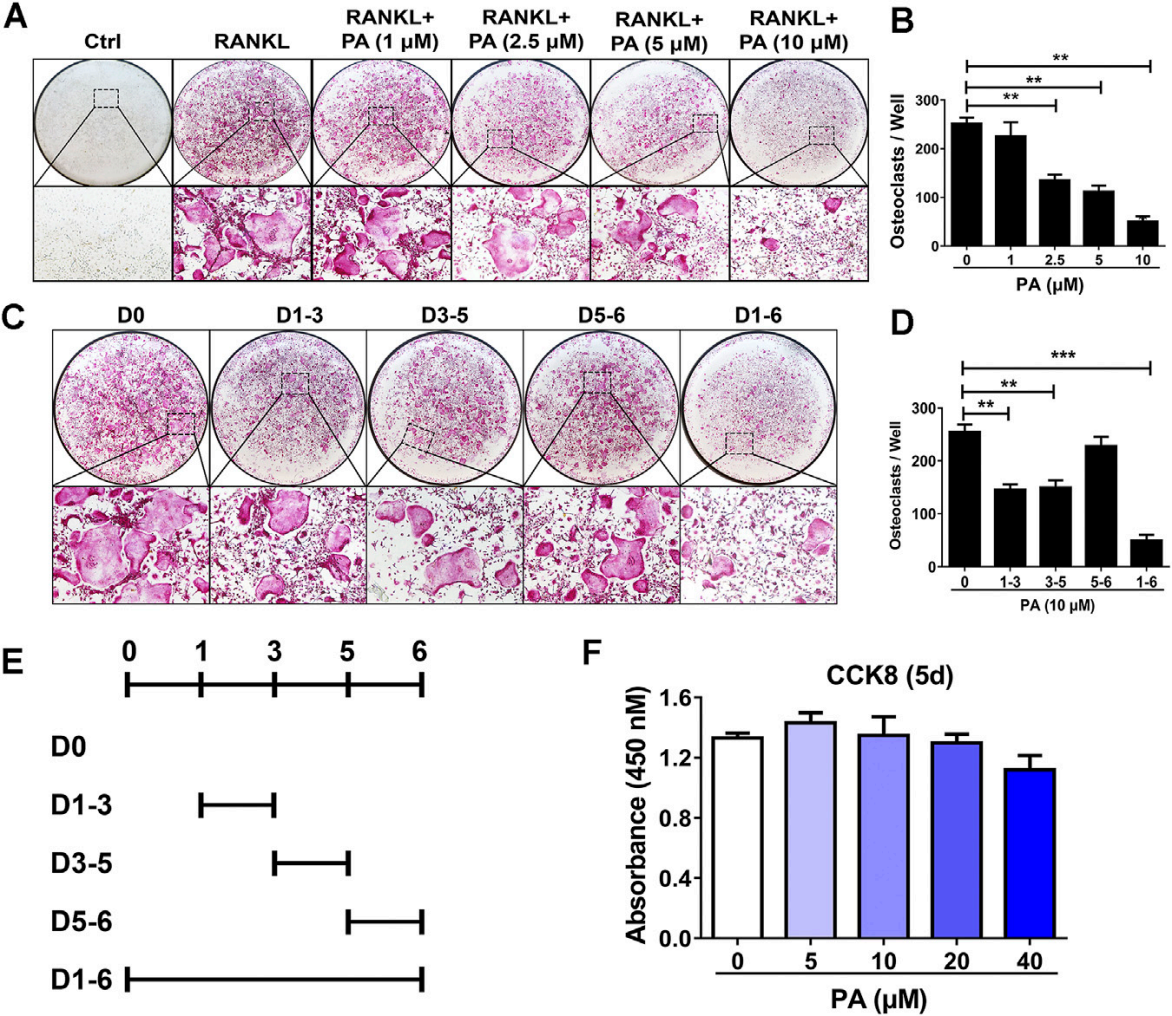
Osteoclastogenesis was impaired by PA. **(A)** Representative images of TRAcP-positive cells after stimulation with PA at different on the indicated days (magnification = ×100). **(B)** TRAcP-positive cells in 96-well plates stimulated with PA at different concentrations on the indicated days were counted and analyzed **(C, E)** Representative images of TRAcP-positive cells after stimulation with 10 μM PA on the indicated days (magnification = ×100). **(D)** TRAcP-positive cells in 96-well plates stimulated with 10 μM PA on the indicated days were counted and analyzed. **(F)** A CCK-8 assay was performed to detect the cytotoxicity of PA against BMMs.

To examine the effects of PA on osteoclastogenesis, experiments to evaluate its inhibitory effect on OC formation were performed. After treatment with 50 ng/ml RANKL for 5 days, PA from 1 mg/ml to 10 mg/ml inhibited OC formation in a dose-dependent manner. PA at a concentration of 10 μg/ml had the most pronounced inhibitory effect (Figures 1A,B). TRAcP staining revealed that the number and size of TRAcP-positive multinucleated OCs were significantly reduced upon PA treatment (Figure 1A). However, there was no striking difference in absorbance between the groups with or without PA treatment, which indicates that PA at concentrations lower than 40 μM exerted no cytotoxicity against BMMs (Figure 1F).

Based on the above experiments, we further examined the timepoints at which PA suppresses OC differentiation. BMMs treated with RANKL were exposed to PA during four intervals: days 1–3, days 3–5, days 5–6, and days 1–6 (Figure 1E). The inhibitory effect of PA on the BMMs was primarily observed early in differentiation and in the middle of differentiation (days 1–3, days 3–5), and this inhibitory effect had weakened by days 5–6 (Figures 1C,D). These findings show that PA inhibited the differentiation of RANKL-induced OCs over the entire process in a dose-dependent manner with no significant cytotoxicity.

### Patchouli Alcohol Affected Bone Resorption

**FIGURE 2:**
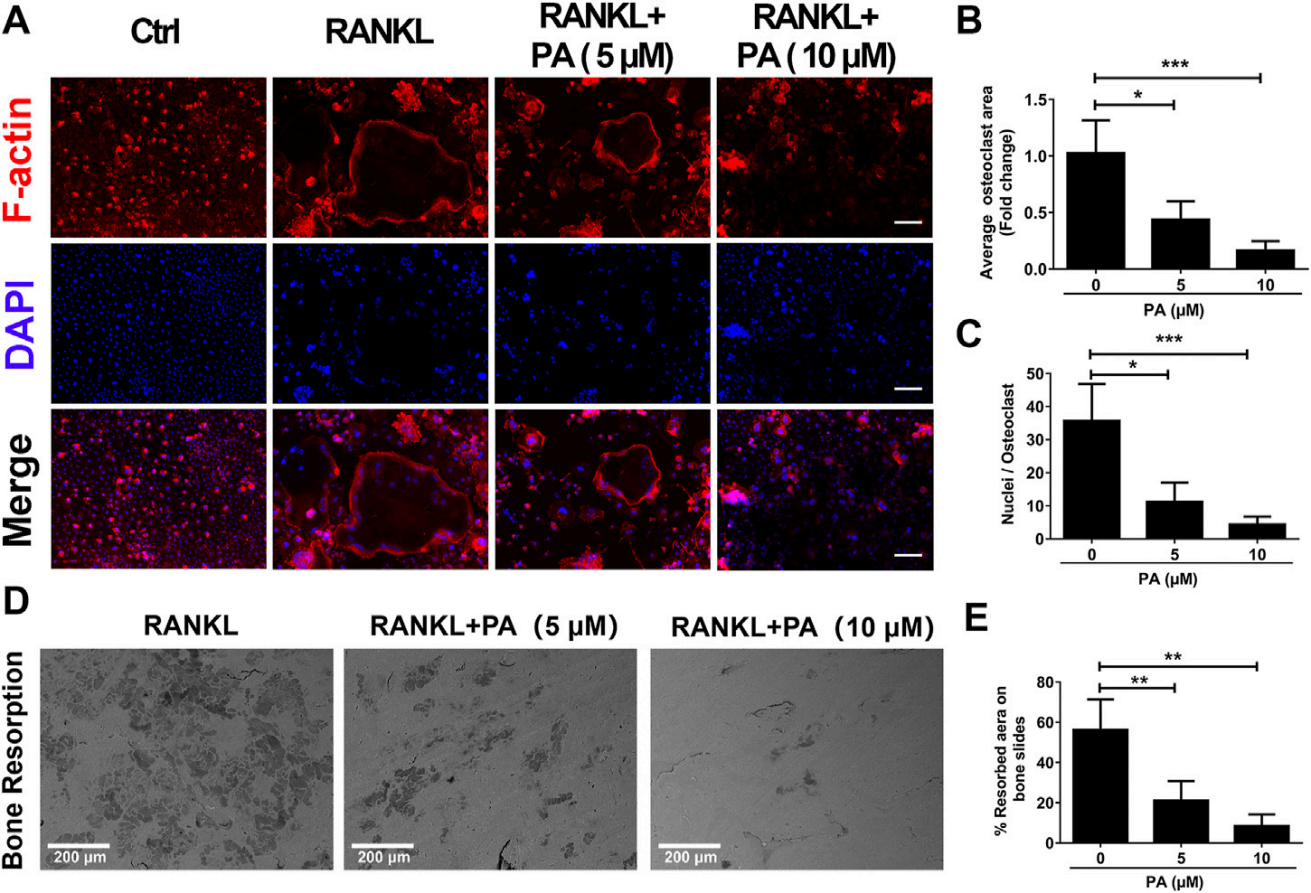
Bone resorption was impaired by PA. **(A)** Representative confocal images of F-actin and nuclei in OCs treated with or without 5 μM or 10 μM PA and subjected to immunofluorescence staining (scale bar = 200 μm). **(B, C)** Quantification of the average OC area and mean nuclear number in OCs. **(D)** Representative images of the bone resorption area in bone slices after OCs were treated with RANKL in the presence or absence of 5 μM or 10 μM PA (scale bar = 200 μm). **(E)** Quantification of the bone resorption area in bone slices.

To evaluate the function of OCs, experiments to test bone resorption were performed. F-Actin belt formation is key for OCs to dissolve bone. Mature OCs treated with RANKL had a large number of clear F-actin belts and numerous nuclei (Figure 2A). After PA treatment, the size and nucleation of the cells decreased, and the number and distribution of F-actin belts were drastically decreased. The inhibitory effect of 10 μM PA was more pronounced (Figures 2A–C). Mature OCs were cultured on bone plates for 2 days in the presence of 5 and 10 μM PA, and the demineralized area in each well was measured and compared to that in a control group (Figures 2D,E). Compared to that in the control group, the resorption area on the bone plate was dramatically reduced in the PA groups, and the demineralized area in the group treated with 10 μM PA was reduced to a greater extent than that in the group treated with 5 μM PA. Therefore, PA dose-dependently impaired bone absorption, and 10 μM PA was significantly effective.

### Patchouli Alcohol Inhibited the Activation of Receptor Activator of Nuclear Factor Kappa-B Ligand-Induced OC Marker Genes

**FIGURE 3:**
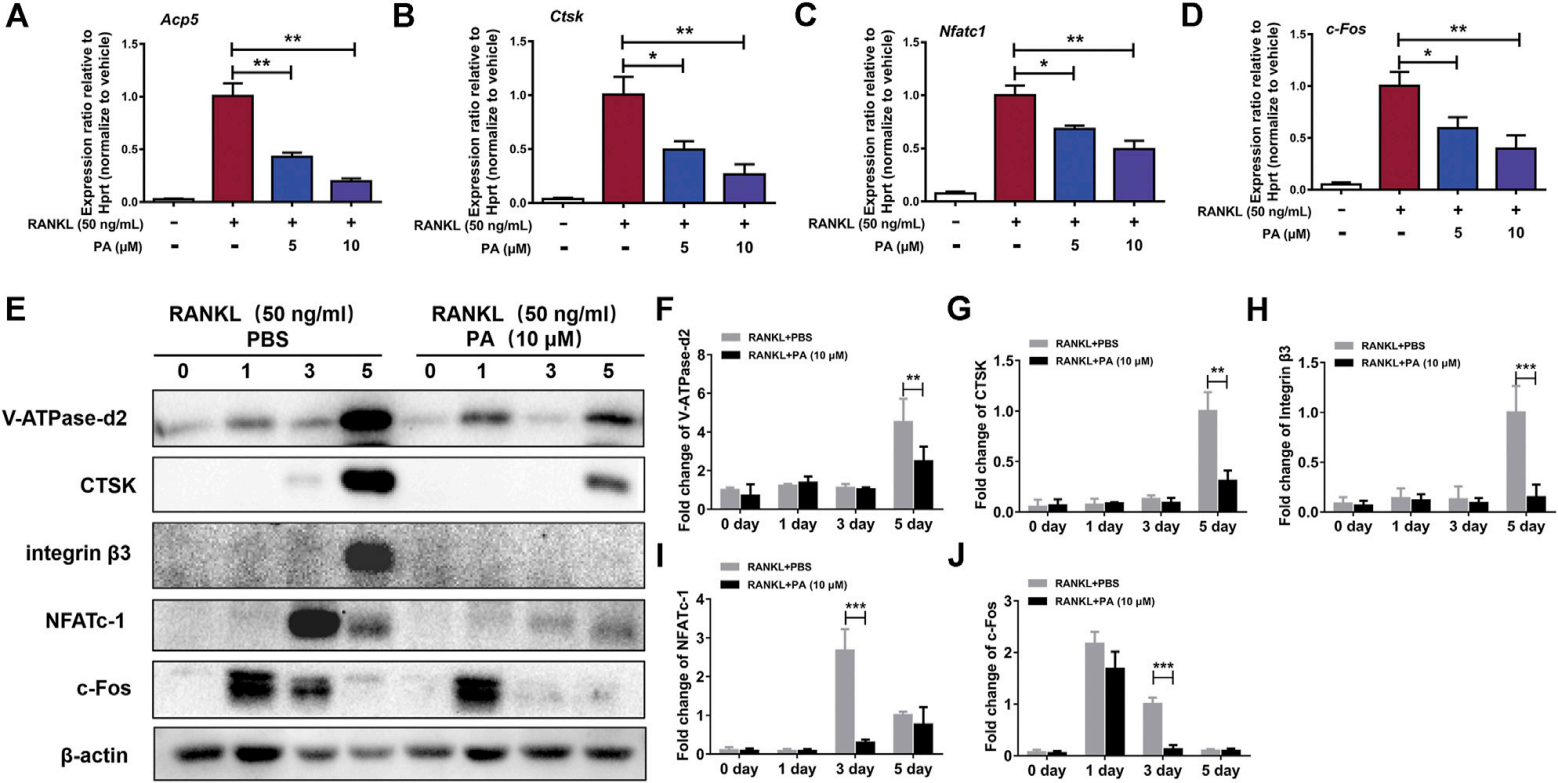
PA inhibited RANKL-induced OC marker genes. **(A–D)** Real-time PCR was used to detect the mRNA expression levels of tartrate-resistant acid phosphatase (TRAcP/acp5), cathepsin K (Ctsk), c-Fos, and nuclear factor of activated T cells, cytoplasmic 1 (NFATc1) in the presence or absence of 5 μM or 10 μM PA. All of the data were normalized to data for the housekeeping gene Hprt. **(E)** Western blotting was performed to measure the protein content of V-ATPase-d2, CTSK, integrin β3, NFATc1, and c-Fos at day 0, day 1, day 3, and day 5 after stimulation with GST-rRANKL (50 ng/ml) with or without PA (10 μM). **(F–J)** Quantitative analysis of protein expression in the PA-stimulated and control groups at different times. The expression of all proteins was normalized to β-actin expression. The data represent the mean ± SD. Significant differences are indicated as follows: *p < 0.05, **p < 0.01, and ***p < 0.001.

To confirm the effects of PA on RANKL-induced OC formation and function, the levels of relevant OC marker genes and expression levels of critical proteins were tested. qRT-PCR was performed to detect the levels of various key genes, including Acp5 (TRAcP), CTSK, NFATc1, and c-Fos. The expression levels of these genes were remarkably reduced after stimulation with PA (10 μM) (Figures 3A–D). Compared to their levels in the control group, PA downregulated the bone resorption-related proteins CTSK, vacuolar (H)-ATPase-d2 (V-ATPase-d2), and integrin β3 and inhibited the expression of NFATc1 and c-Fos on day three (Figures 3E–J). These results further confirm the inhibitory effects of PA on RANKL-induced OC formation and function.

### Patchouli Alcohol Suppressed Receptor Activator of Nuclear Factor Kappa-B Ligand-Induced NF-κB Signaling

**FIGURE 4:**
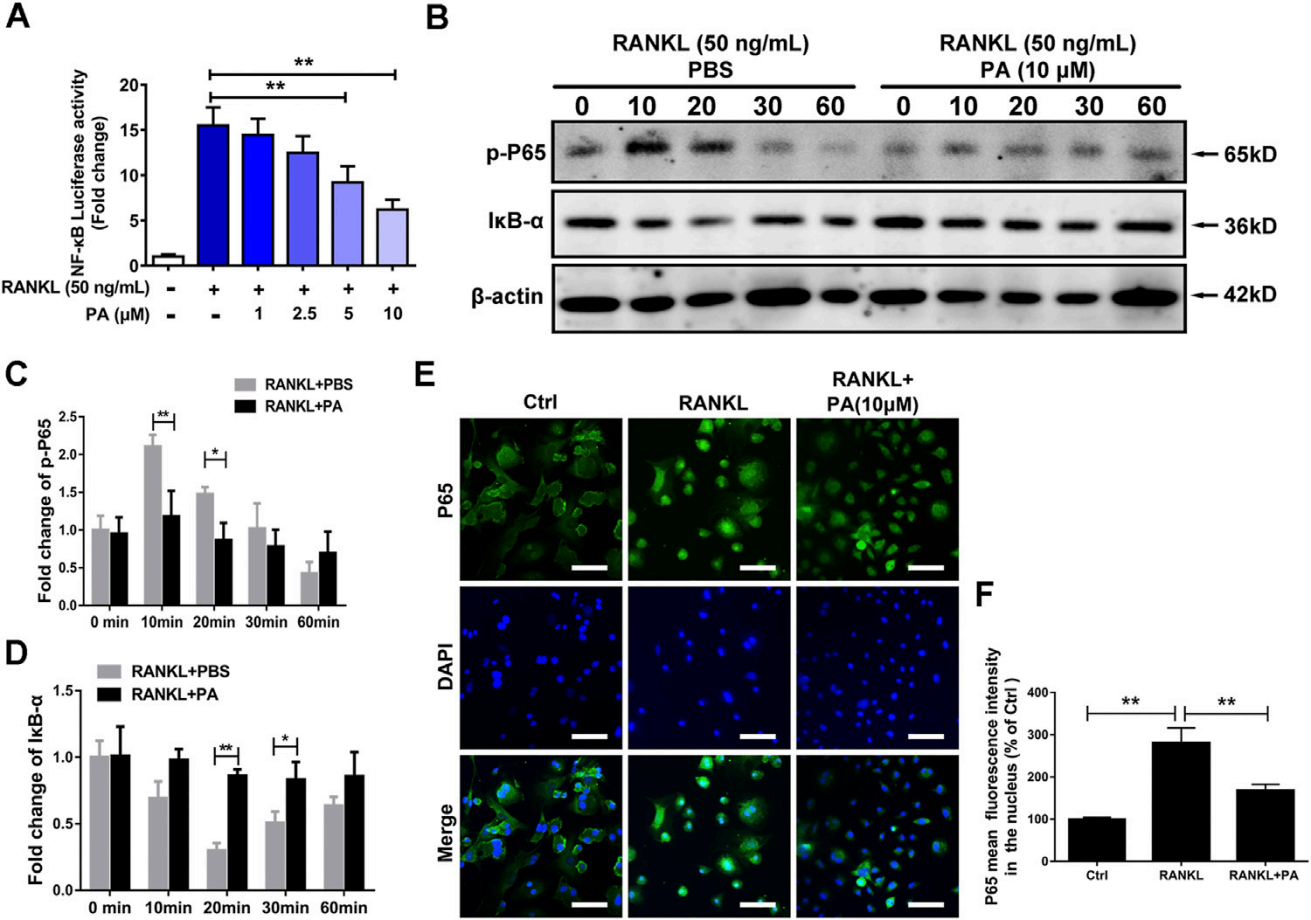
PA suppressed RANKL-induced NF-κB signaling. **(A)** NF-κB luciferase activity in BMMs treated with PA at different concentrations. **(B)** Western blotting was performed to measure the protein content of p-P65 and IκB-α after 0, 10, 20, 30, and 60 min of stimulation with GST-rRANKL (50 ng/ml) with or without PA (10 μM). **(C,D)** Quantitative analysis of protein expression in the PA-stimulated and control groups at different times. The expression of all proteins was normalized to β-actin expression. **(E)** Representative confocal images of p65 in OCs treated with or without 10 μM PA subjected to immunofluorescence staining (scale bar = 200 μm). The nuclear translocation of p65 is indicated in this figure. **(F)** Quantitative analysis of the mean p65 fluorescence intensity in the nucleus after sample processing as described in **(E)**.

To examine the underlying mechanism and to discover the relationship between PA and NF-κB signaling pathways, luciferase experiments were performed. Compared to that in the RANKL group, PA had a significant inhibitory effect on the transcriptional activity of NF-κB, as shown via analyses of luciferase activity (Figure 4A). To examine the specific mechanism, we further tested the protein expression of p65 and IκB-a. The data showed that PA upregulated the expression of IκB and inhibited the phosphorylation of p65 (Figures 4B–D). Therefore, to examine the effects of changes in IκB and p-p65 on the nuclear translocation of NF-κB, immunofluorescence experiments were performed. Compared to those in the RANKL group, the number of puncta containing the p65 protein in the nucleus were significantly decreased after PA administration (Figure 4E), which was further verified by the decrease in mean p65 fluorescence intensity (Figure 4F). Therefore, PA suppressed NF-κB nuclear translocation via inhibition of p65 phosphorylation and upregulation of IκB.

### Patchouli Alcohol Activated Its Target, PXR, to Suppress Downstream NF-κB Signaling

**FIGURE 5:**
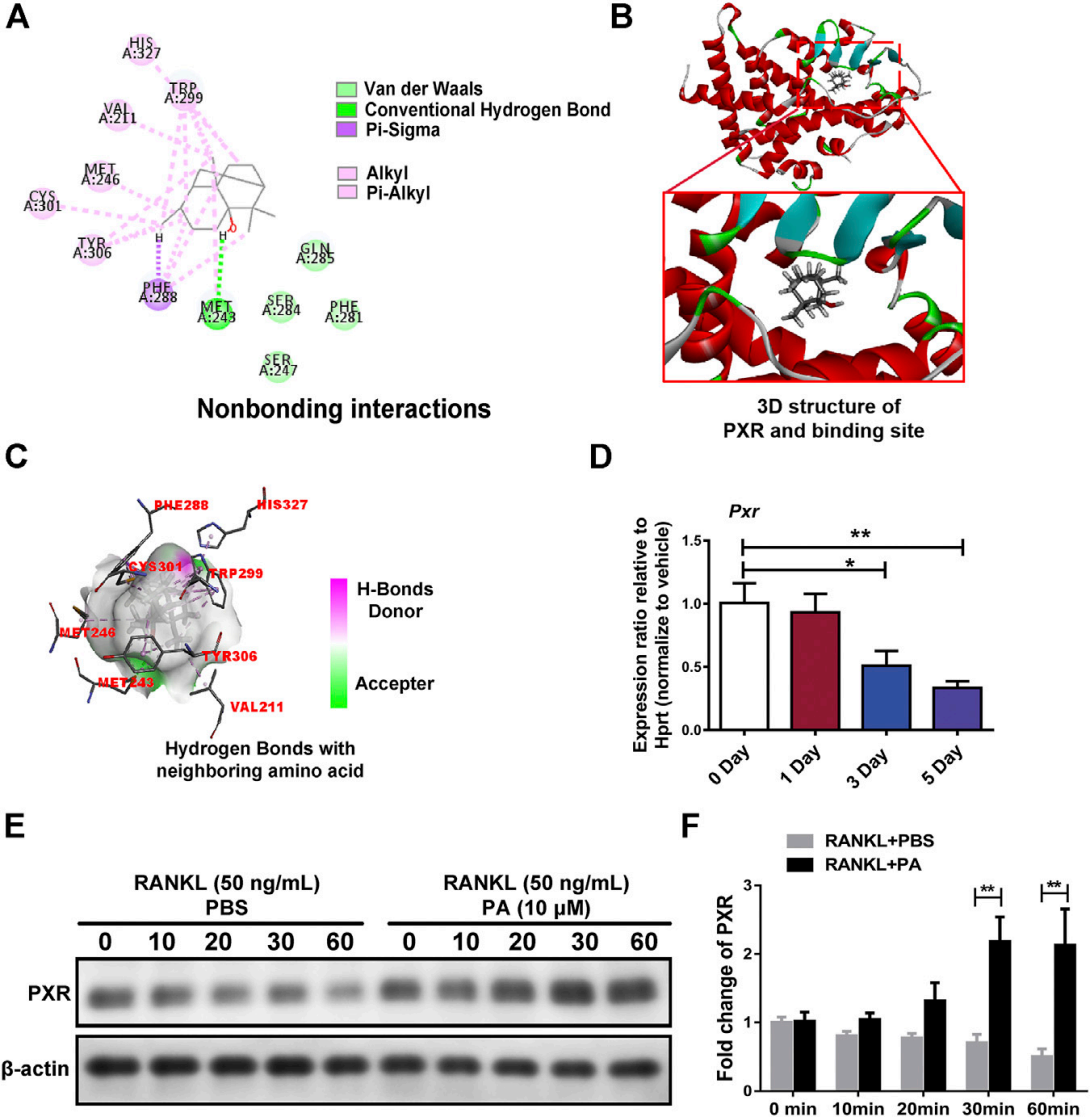
PA activated PXR. **(A)** Nonbonding interactions between PA and PXR (Compound CID of PA: 10955174). **(B)** 3D structure of PXR and its binding site for PA. **(C)** Hydrogen bonds between PA and neighboring amino acid residues in PXR. **(D)** Real-time PCR was used to detect the mRNA expression levels of PXR after RANKL-induced OC differentiation for different durations. All data were normalized to data for the housekeeping gene Hprt. **(E)** Western blotting was performed to measure the protein content of PXR after 0, 10, 20, 30, and 60 min of stimulation with GST-rRANKL (50 ng/ml) with or without PA (10 μM). **(F)** Quantitative analysis of PXR protein expression in the PA-stimulated and control groups at different times. The expression of all proteins was normalized to β-actin expression.

Target prediction directed our focus to PXR, the expression of which was decreased in OCs upon PA treatment (Figure 5D). As shown in Figures 5A,B high-affinity binding site between PA and PXR was obtained by analysis with Discovery Studio. Furthermore, nonbonding interactions between PA and PXR at this site, including intermolecular forces and conjugated systems, were identified (Figure 5A). The presence of hydrogen bonds was found to drastically enhance the binding affinity between PXR and PA (Figure 5C), which suggests that PA and PXR specifically interact. The prototype PXR ligand pregnenolone 16α-carbonitrile (PCN) was applied to make comparison. Based on the high-affinity binding site between PCN and PXR (Supplementary Figure S1A), the nonbonding interaction of PCN is found no significant differences compared with PA, including hydrogen bonds (Supplementary Figures S1B–C). When the isomery of PA were used for molecular docking, the results suggest the high binding affinity with PXR, featuring with the presence of hydrogen bonds (Supplementary Figures S2–S4). Therefore, PXR is predicted to be the potential target of PA.

**FIGURE 6:**
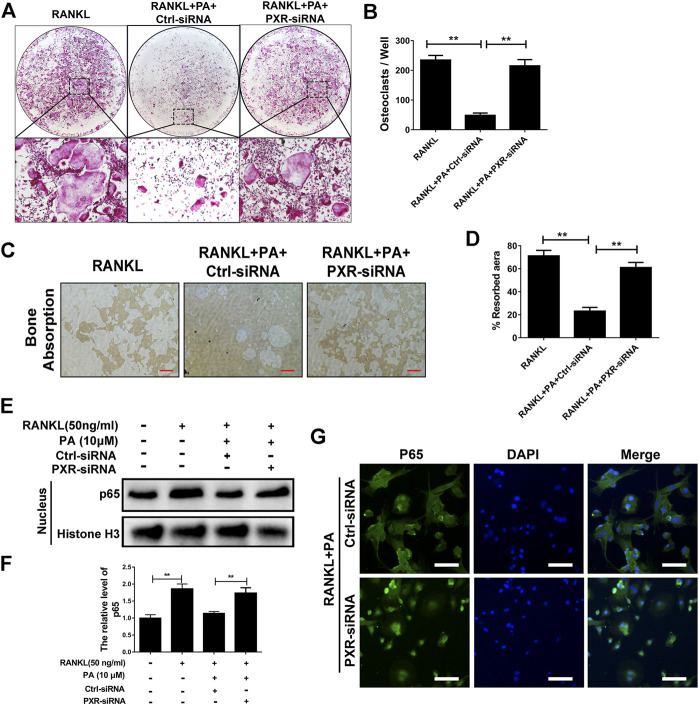
PA activated its target, PXR, to suppress downstream NF-κB signaling. **(A)** Representative images of TRAcP-positive cells after transfection with siRNAs in the presence of PA (10 μM) (magnification = ×100). **(B)** TRAcP-positive cells transfected with siRNAs in the presence of PA (10 μM) in 96-well plates were counted and analyzed. **(C)** Representative images of the bone resorption area on hydroxyapatite-coated plates upon transfection with siRNAs in the presence of PA (10 μM) (magnification = ×100). **(D)** Quantification of the bone resorption area on hydroxyapatite-coated plates. **(E)** Western blotting was performed to measure the p65 protein content in the nucleus after transfection with siRNAs in the presence of PA (10 μM) for 1 h. **(F)** Quantitative analysis of p65 protein expression in the nucleus after transfection with siRNAs in the presence of PA (10 μM) for 1 h. The expression of all proteins was normalized to histone H3 expression. **(G)** Representative confocal images showing p65 after transfection with siRNAs in the presence of RANKL and PA (10 μM) under immunofluorescence staining (scale bar = 200 μm).

Upon stimulation with 10 μM PA, the expression of PXR increased remarkably (Figures 5E,F), which indicates that PA is an agonist of PXR. To confirm the primary role of PA, PXR-siRNA was used. TRAcP staining and bone absorption analysis showed that the number of OCs among RANKL-induced BMMs transfected with PXR-siRNA and resorption area upon PA stimulation were similar to those in the group treated with only RANKL, but the ctrl-siRNA group showed normal inhibition of these parameters (Figures 6A–D). These results show that PA activated PXR and counteracted the effects of PXR on osteoclastogenesis and bone resorption in the presence of PXR-siRNA.

To identify whether the inhibitory effect of PA on NF-κB nuclear translocation was mediated by PXR, p65 protein expression and nuclear translocation were examined. We observed almost normal p65 expression (Figures 6E,F) and a large amount of nuclear-translocated p65 (Figure 6G) in PA-treated BMMs transfected with PXR-siRNA. Therefore, PA inhibits NF-κB nuclear translocation by stimulating PXR.

### Patchouli Alcohol Downregulated TLR4/Myd88/TRAF6 *via* PXR

**FIGURE 7:**
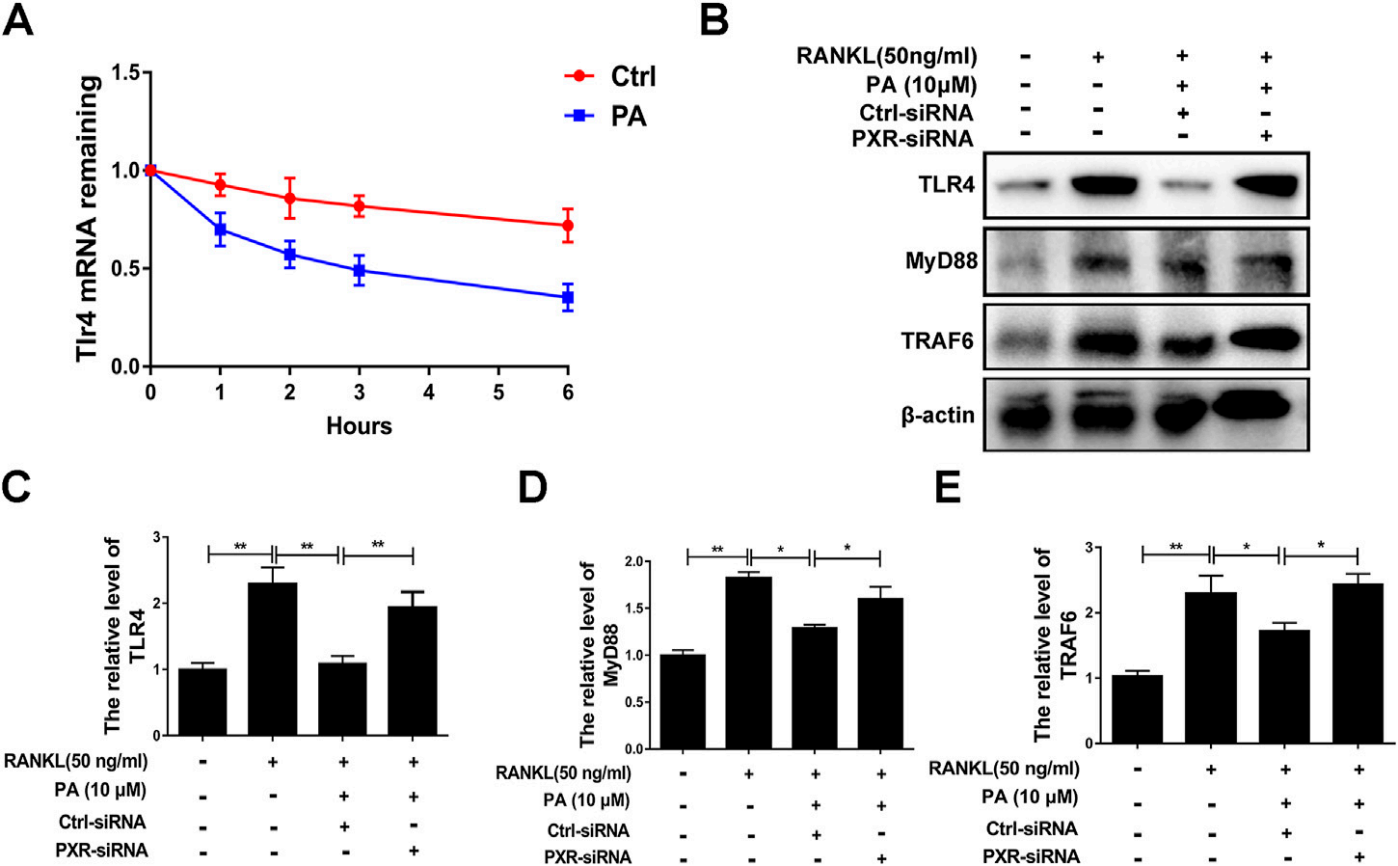
PA downregulated the TLR4/Myd88/TRAF6 axis. **(A)** The fold change in TLR4 mRNA expression after treatment with or without PA for 0, 1, 2, three or 6 h determined using real-time PCR. **(B)** Western blotting was performed to measure the TLR4, MyD88, and TRAF6 protein content after transfection with siRNAs in the presence of PA (10 μM) for 1 h. **(C–E)** Quantitative analysis of TLR4, MyD88, and TRAF6 protein expression after transfection with siRNAs in the presence of PA (10 μM) for 1 h. The expression of all proteins was normalized to β-actin expression.

A previous study demonstrated that TLR4 negatively regulates PXR and TLR4, and its downstream adapter, Myd88, was found to be closely related to the recruitment of TRAF6. In PA-treated BMMs transfected with ctrl-siRNA, PA decreased the protein levels of TLR4, Myd88 and TRAF6, but this inhibitory effect was reduced in BMMs transfected with PXR-siRNA (Figures 7B–E). PA suppressed the TLR4/Myd88/TRAF6 axis *via* PXR activation. However, understanding the detailed relationship between PXR and TLR4 required further discovery. We found that the TLR4 mRNA content decreased significantly within 1 h of PA treatment (Figure 7A). Based on the data in Figure 7A, the half-life of TLR4 mRNA was shortened after PA administration, which further indicated that TLR4 stability was inhibited by PA. TRAF6 recruitment is critical for NF-κB nuclear translocation. Therefore, in combination with the results of previous experiments, these findings confirmed that PA inhibited NF-κB nuclear translocation through PXR *vi*a suppression of the TLR4/Myd88/TRAF6 axis.

### Patchouli Alcohol Attenuated OVX-Induced Bone Loss *In Vivo*

Animal experiments were performed to further assess the role of PA in the treatment of bone-soluble diseases. OVX model mice were treated with vehicle (NS) or PA every 2 days for 6 weeks and then subjected to micro-CT and histomorphometric assessments. The obtained bone parameters were used to determine the effects of PA on bone mass maintenance.

**FIGURE 8:**
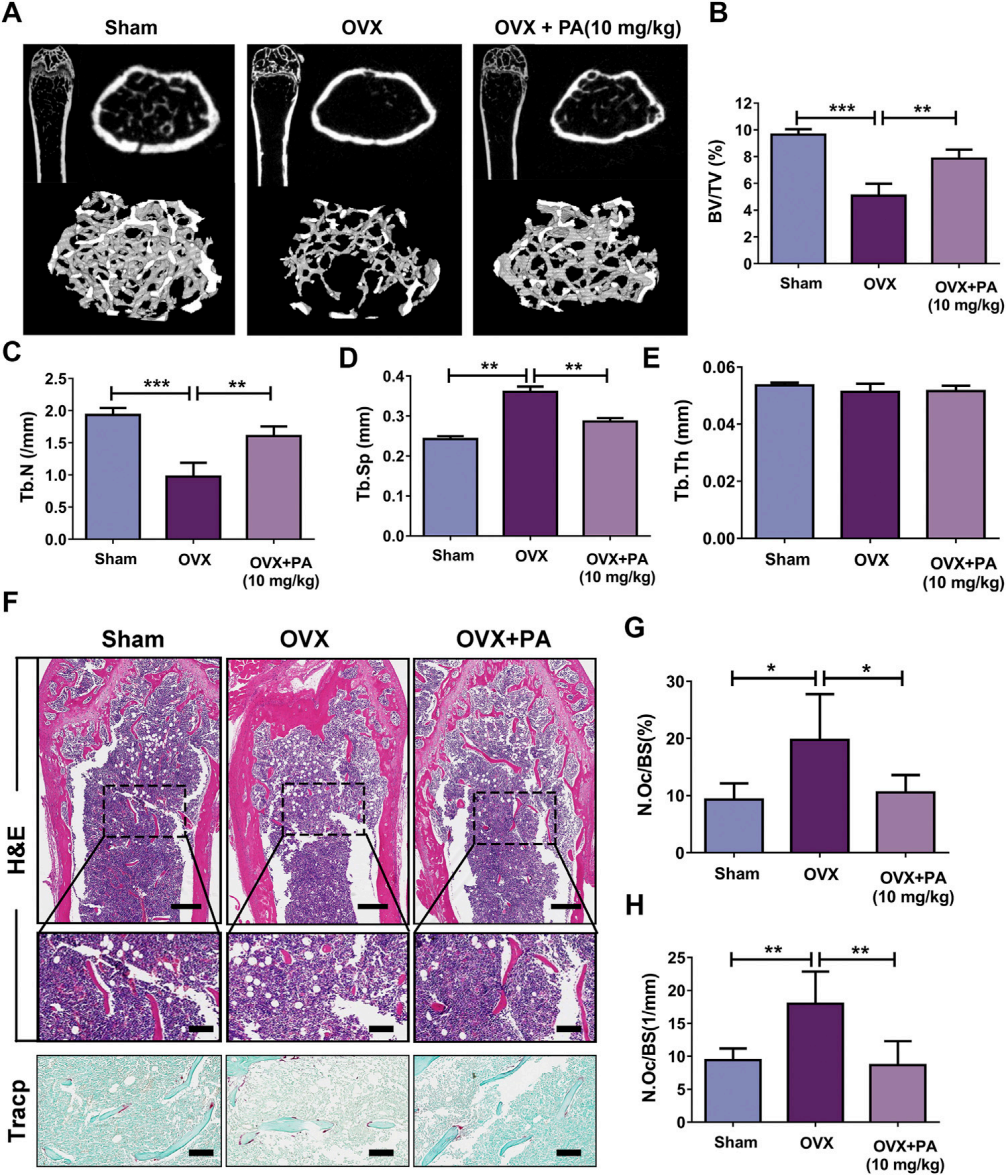
PA inhibited OCs in the OVX mouse model. **(A)** 3D computer reconstruction of the femur and tibias in each group captured by a micro-CT instrument. **(B–E)** The relevant bone microstructure-related parameters were quantitatively measured: BV/TV, Tb. Sp., Tb. N, and Tb. Th. **(F)** Representative images of tibias stained with H and E and TRAcP. Scale bar = 500 mm; scale bar = 100 mm in the enlarged pictures. **(G, H)** Quantitative analyses of the OC number per bone surface (N.Oc/BS).

The micro-CT results revealed that compared to those in the vehicle group (Figure 8A), BV/TV (Figure 8B) and Tb. N (Figure 8C) were remarkably elevated, while Tb. Sp (Figure 8D) was declined upon PA treatment, showing that bone loss was significantly inhibited by PA. No statistically significant difference in Tb. Th was found between the groups, indicating that the thickness of the girder was not affected by estrogen deficiency (Figure 8E).

Histomorphometric assessments were performed to further verify the results. The bone evaluation parameter N. Oc/BS decreased significantly after treatment with PA (*p* < 0.05 compared to that in the OVX group), as shown by H and E and TRACP staining, which suggests that the number of OCs *in vivo* was drastically decreased. In summary, these results show that PA has a protective effect against bone loss resulting from estrogen deficiency.

## Discussion

The present study demonstrated that PA inhibited osteoclastogenesis *in vitro* by downregulating the NF-κB signaling pathway via activation of its target, PXR. Furthermore, PA attenuated OVX-induced *in vivo* bone loss by suppressing the formation of OCs. These findings reveal that PA is a promising candidate drug for the treatment of osteoporosis.

In an *in vitro* model, PA dose-dependently inhibited RANKL-induced OC differentiation and bone resorption. PA downregulated master regulators of OC differentiation, including NFATc1 and other OC-related genes (Takayanagi et al., 2002). During the RANKL-induced OC differentiation process, TRAF6 is a direct medium through which the classical NF-κB pathway is promoted after the binding of RANK and RANKL, which ultimately affects the nuclear transfer of p65 (Tan et al., 2017). PA inhibited the degradation of IκB-α and p65 phosphorylation in our study, which suggests that PA suppresses the nuclear translocation of p65. This hypothesis was supported by the results of immunofluorescence staining. The results reveal that RANKL-induced NF-κB signaling is involved in the inhibitory effect of PA on osteoclastogenesis.

Previous studies showed that PA, NF-ĸB signaling and PXR are involved in the inflammatory reaction (Lawrence 2009; Jeong et al., 2013; Okamura et al., 2020). Although PA has been reported to activate PXR, this effect has not been reported in OCs (Zhang et al., 2020). In our study, in addition to the experimental evidence, molecular docking showed the influence of PA isomers and suggested that PA binds to PXR. To determine the specific relationship between PA and PXR in OCs, we used semiflexible molecular docking. In the experiment, the receptor was subjected to rigid docking, and PA was subjected to flexible docking to consider the possible docking of different isomers of PA. Finally, molecular docking analysis demonstrated that PA is likely a target of PXR. For further verification, different PA isomers were directly used for molecular docking, and the results of all isomers suggested their strong binding affinity with PXR (Supplementary Figures S2–S4) More specifically, other isomers of PA (CID: 442384; CID: 6432585; CID: 521903) were found interaction with PXR via Hydrogen Bond similar to isomer of PA (CID: 10955174). However, original isomer (CID: 10955174) exerts more nonbonding interaction like Pi-Sigma than others. Although moledule docking indicates the original isomer of PA (CID: 10955174) earn an edge, the isomery of PA shows no significant difference as for its interaction with PXR. Interestingly, moledule docking was also applied to analyze the relationship between ligand and PXR (Shao et al., 2021), but compared with their study, we have take more nonbonding interactions into consideration, including Pi-Sigma, Pi-Alkyl, Van der Waals, which further indicating the high binding affinity of PA and PXR. Our results also showed that PA-mediated inhibition of NF-κB nuclear translocation was reversed by PXR-siRNA transfection, which suggests that PXR is key to the effects of PA on the NF-κB signaling pathway in OCs. However, how these pathways are linked and their function in regulating osteoclastogenesis are not known.

Our research showed that PA negatively regulates the TLR4 mRNA level via activation of PXR, which inhibits the recruitment of TRAF6 and nuclear translocation of p65. According to a previous study, the negative regulatory effect of PXR on TLR4 primarily involves of its inhibitory effect on TLR4 mRNA stability (Huang et al., 2018). In our experiment, the stability of TLR4 was drastically inhibited in the initial period after PXR activation, and the half-life of TLR4 declined remarkably. Levels of the TLR4 downstream adapter Myd88 (Ve et al., 2017) and recruited TRAF6 (He et al., 2016) returned to normal levels after PXR-siRNA treatment, which indicates that PA downregulates the TLR4/Myd88/TRAF6 signaling cascade via PXR, ultimately inhibiting the nuclear translocation of NF-κB.

Interestingly, although our experiments proved that PA can act as an agonist of PXR and upregulate the expression of PXR, in contrast, PXR agonists, including PCN, rifampicin, and SR-12813, in fact downregulate PXR mRNA (Bailey et al., 2011; Smutny et al., 2020). Notably, PCN at high concentrations reduced the transcription level of PXR mRNA, but the expression of PXR target genes was not significantly inhibited and in fact high (Bailey et al., 2011).One possible explanation for this finding is that nuclear receptors carry out self- and cross-regulation mechanisms (Bagamasbad and Denver 2011). Under agonist treatment, excess PXR can inhibit self-transcription and promote the degradation of self-mRNA by activating miR-18a-5p, which ultimately leads to the suppression of PXR transcription (Bailey et al., 2011). Therefore, PXR may protect itself through this feedback regulation. This self-protective mechanism was not shown by our experimental results but it is worthy of attention in future experiments.

Despite the complex regulatory mechanisms of nuclear receptor, some of them are significant and to be covered. Taking miRNAs as example: miRNAs modify the function of PXR through regulating corresponding transporters and enzymes, combined with the 3′-UTR of PXR mRNA and targeting other transcription factors, nuclear receptors as well as its dimer partners like RXR (Smutny et al., 2021). Also, the co-drug strategy is also to be studied. It’s reported that developing PXR antagonists as co-drug may decline drug toxicity of PXR agonist as well as drug resistance (Chai et al., 2020).

PA was used in an OVX mouse model to further confirm the role of PA in decreasing bone loss *in vivo*. The results showed that PA increased the BV/TV ratio and Tb.N. and decreased Tb. Sp., which suggests that bone loss was markedly attenuated by PA. Some reports have demonstrated that although OVX mice are widely used to study osteoporosis, this model fails to reflect changes in cortical bone loss (Li et al., 2014; Roberts et al., 2019). Whether PA plays an effective role in the treatment of cortical bone is not clear based on our study, and this gap in knowledge may become an obstruction for its further clinical treatment. In addition, the transportation and absorption of PA and the way in which it affects OCs remain to be studied *in vivo*. The metabolism of PA in gastric acid provides a new direction for us. PA will be transformed into β-patchoulene (β-PAE) in acid which suggested more powerful anti-inflammatory activity than PA (Junren et al., 2021). Studies have reported the use of β-cyclodextrin to encapsulate PA, improving its stability *in vivo* (Xu et al., 2017a), and lactoferrin has been used to deliver PA to inflammatory colonic macrophages that express LRP1, the receptor of lactoferrin (Zhao et al., 2020). Therefore, the application of these materials is expected to provide a mechanism to deliver PA to OCs.

## Conclusion

**FIGURE 9:**
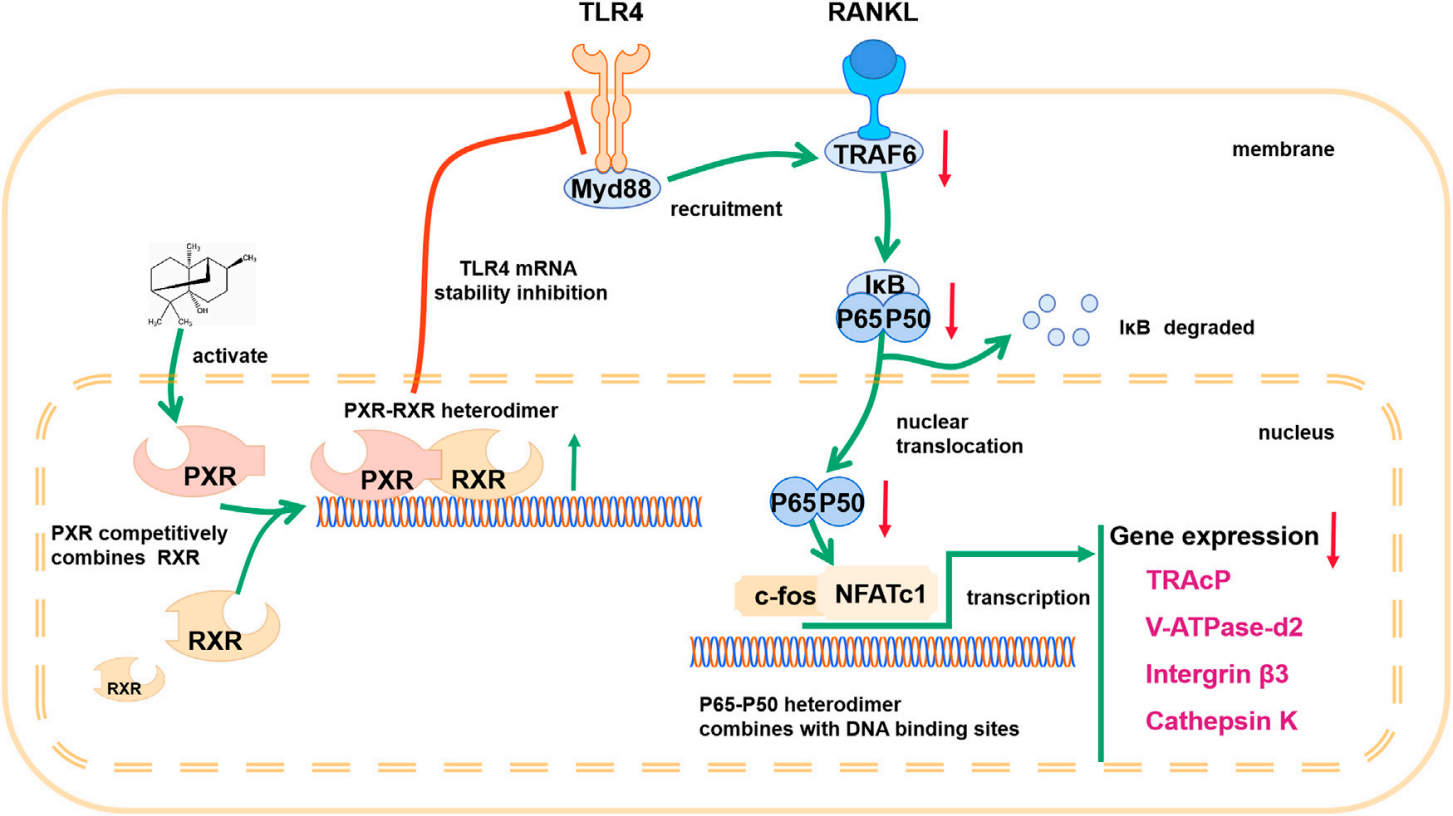
Schematic diagram showing the mechanism by which PA inhibits osteoclastogenesis.

In conclusion, our results show that PA inhibits osteoclastogenesis by negatively regulating activation of the RANKL-induced NF-κB signaling pathway, the main pathway that regulates osteoclastogenesis. Based on its effects on a PXR/NF-κB feedback loop, PA plays a significant role in activating PXR and inhibits the nuclear translocation of p65 (Figure 9) to further regulate the NF-κB signaling pathway. Therefore, these findings suggest that PA can be used in the treatment of OC-related bone diseases in the future.

## Data Availability

The raw data supporting the conclusion of this article will be made available by the authors, without undue reservation.

## Supplementary Material

SUPPLEMENTARY FIGURE 1 Docking result of PCN (CID:15032) and PXR. **(A)** Nonbonding interactions between PCN and PXR. (Compound CID of PCN: 15032). **(B)** 3D structure of PXR and its binding site for PCN. **(C)** Hydrogen bonds between PCN and neighboring amino acid residues in PXR.

SUPPLEMENTARY FIGURE 2 Docking result of PA (CID:442384) and PXR. **(A)** Nonbonding interactions between PCN and PXR. (Compound CID of PA: 442384). **(B)** 3D structure of PXR and its binding site for PCN. **(C)** Hydrogen bonds between PCN and neighboring amino acid residues in PXR.

SUPPLEMENTARY FIGURE 3 Docking result of PA (CID:6432585) and PXR. **(A)** Nonbonding interactions between PCN and PXR. (Compound CID of PA: 6432585). **(B)** 3D structure of PXR and its binding site for PA. **(C)** Hydrogen bonds between PA and neighboring amino acid residues in PXR.

SUPPLEMENTARY FIGURE 4 Docking result of PA (CID:521903) and PXR. **(A)** Nonbonding interactions between PA and PXR. (Compound CID of PA: 521903). **(B)** 3D structure of PXR and its binding site for PA. **(C)** Hydrogen bonds between PA and neighboring amino acid residues in PXR.
