# Supplementary figures and images for: Patchouli Alcohol Modulates the Pregnancy X Receptor/Toll-like Receptor 4/Nuclear Factor Kappa B Axis to Suppress Osteoclastogenesis
Source: Front Pharmacol. 2021 Jun 8;12:684976. doi: 10.3389/fphar.2021.684976 (PMC8227438; doi:10.3389/fphar.2021.684976)

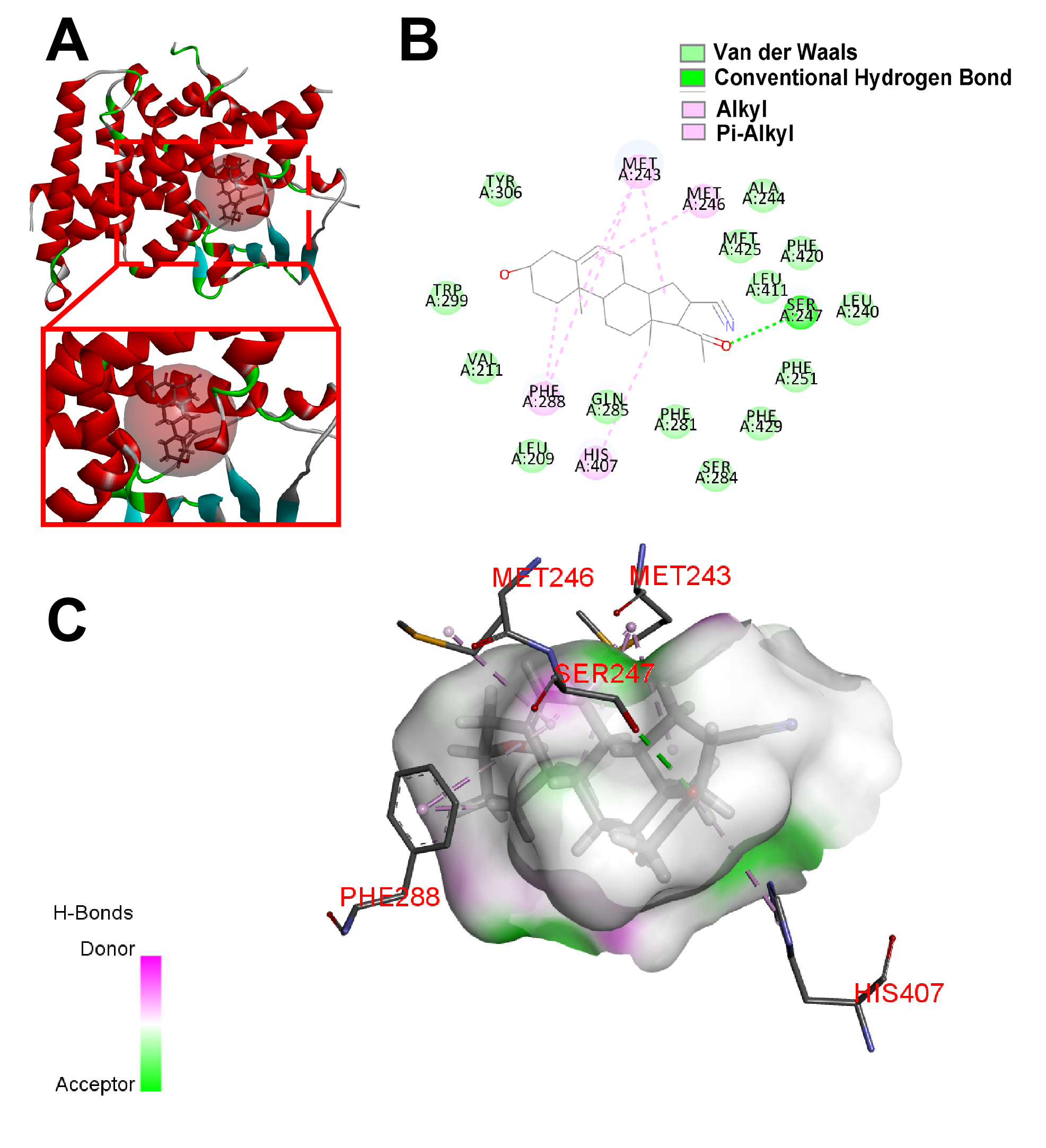

Supplement: Supplementary file 1 [file Image1.TIF]

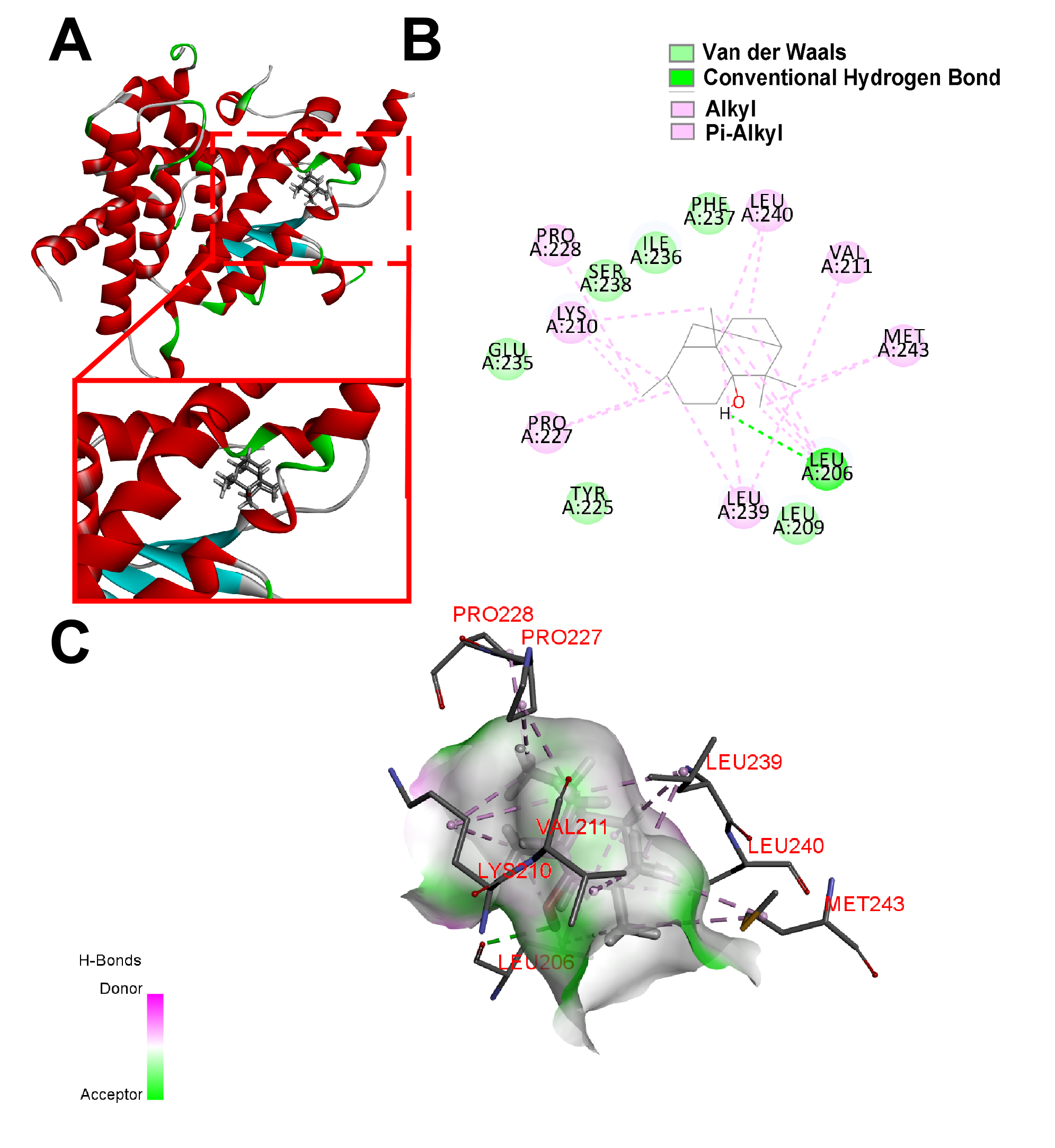

Supplement: Supplementary file 2 [file Image2.TIF]

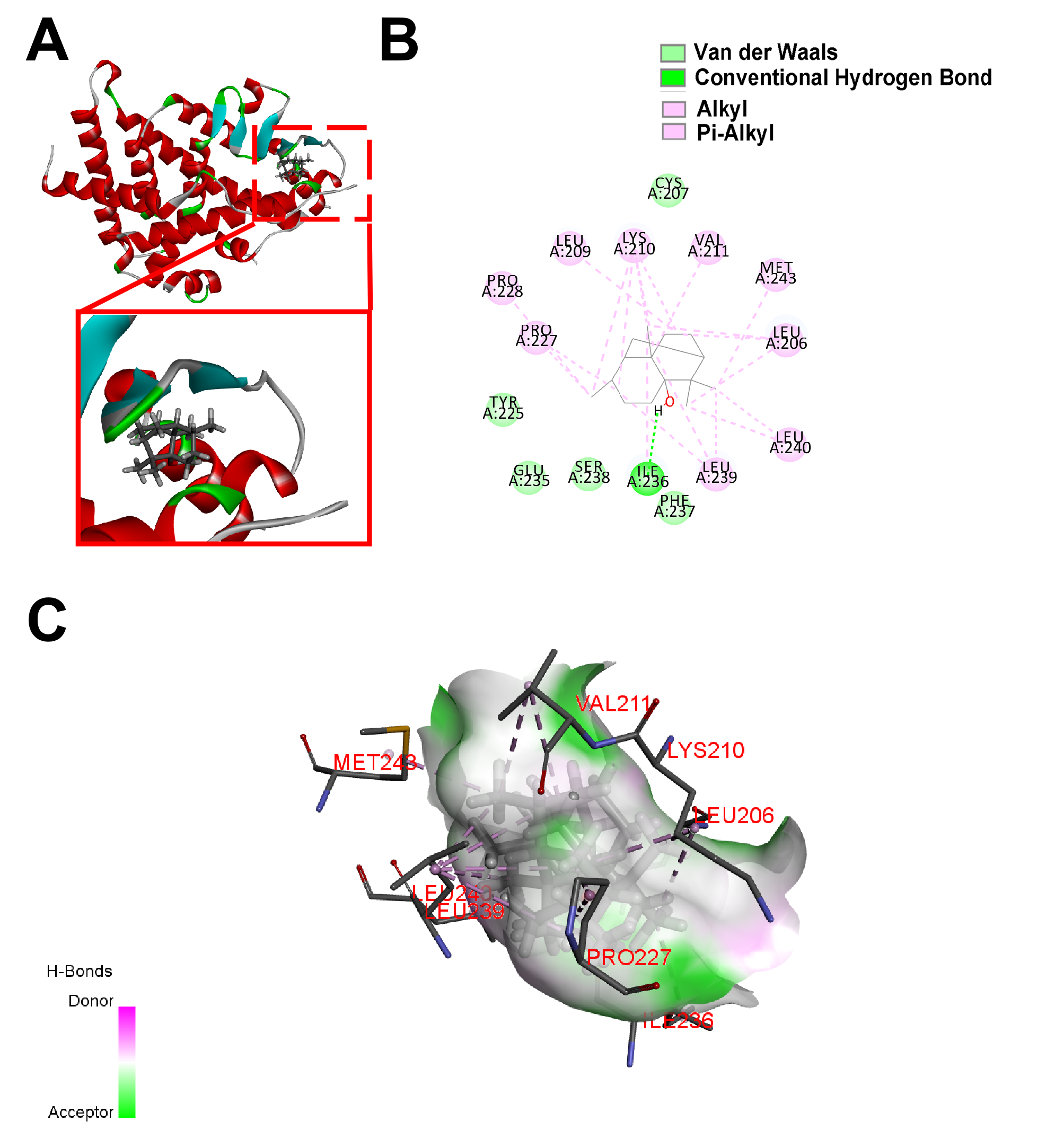

Supplement: Supplementary file 3 [file Image3.TIF]

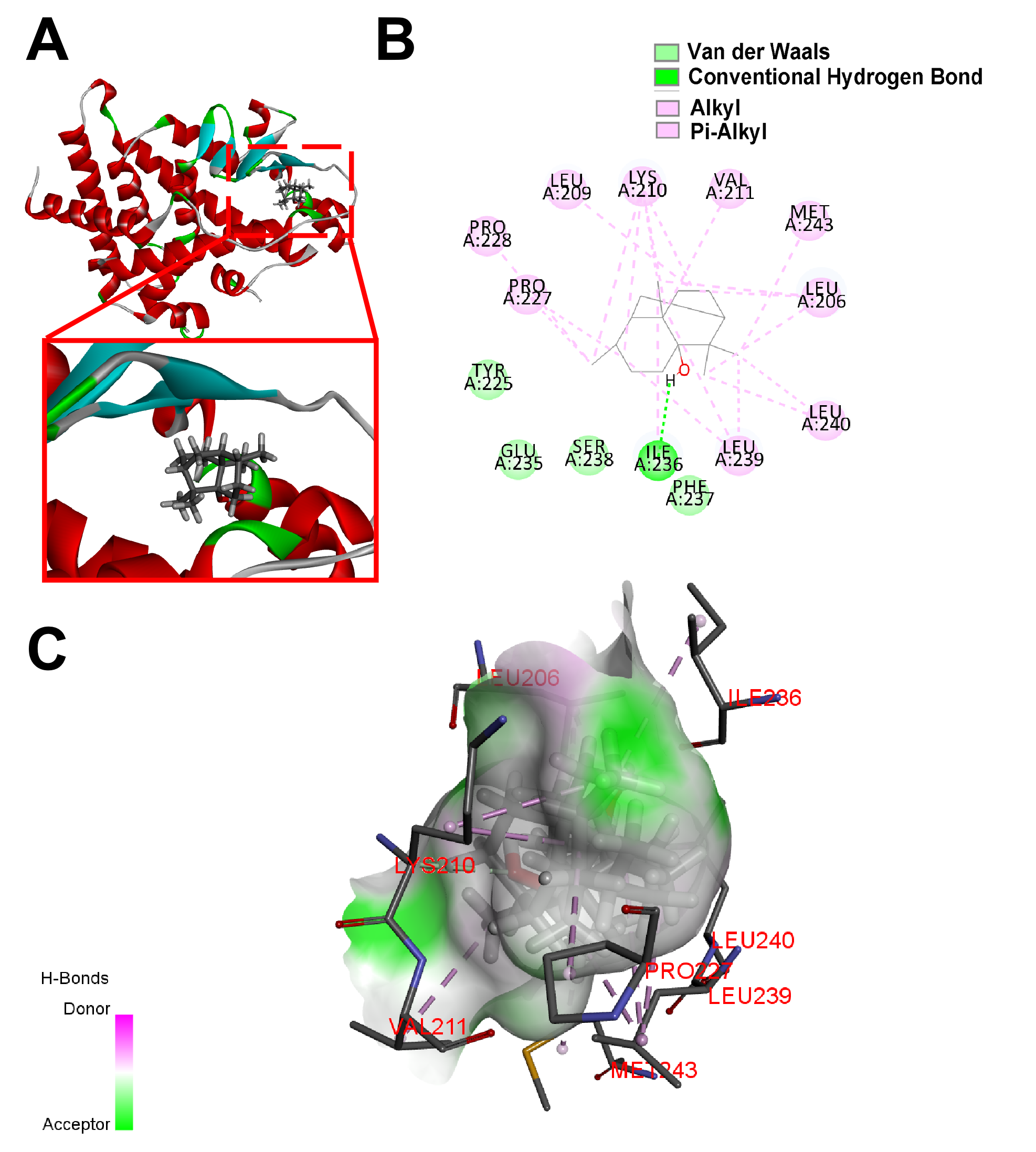

Supplement: Supplementary file 4 [file Image4.TIF]
